# Supplementary material for: Dual-stimuli responsive and reversibly activatable theranostic nanoprobe for precision tumor-targeting and fluorescence-guided photothermal therapy
Source: Nat Commun. 2017 May 19;8:14998. doi: 10.1038/ncomms14998 (PMC5454460; doi:10.1038/ncomms14998)
Supplement: Supplementary Information — Supplementary Figures, Supplementary Methods, and Supplementary References [file ncomms14998-s1.pdf]

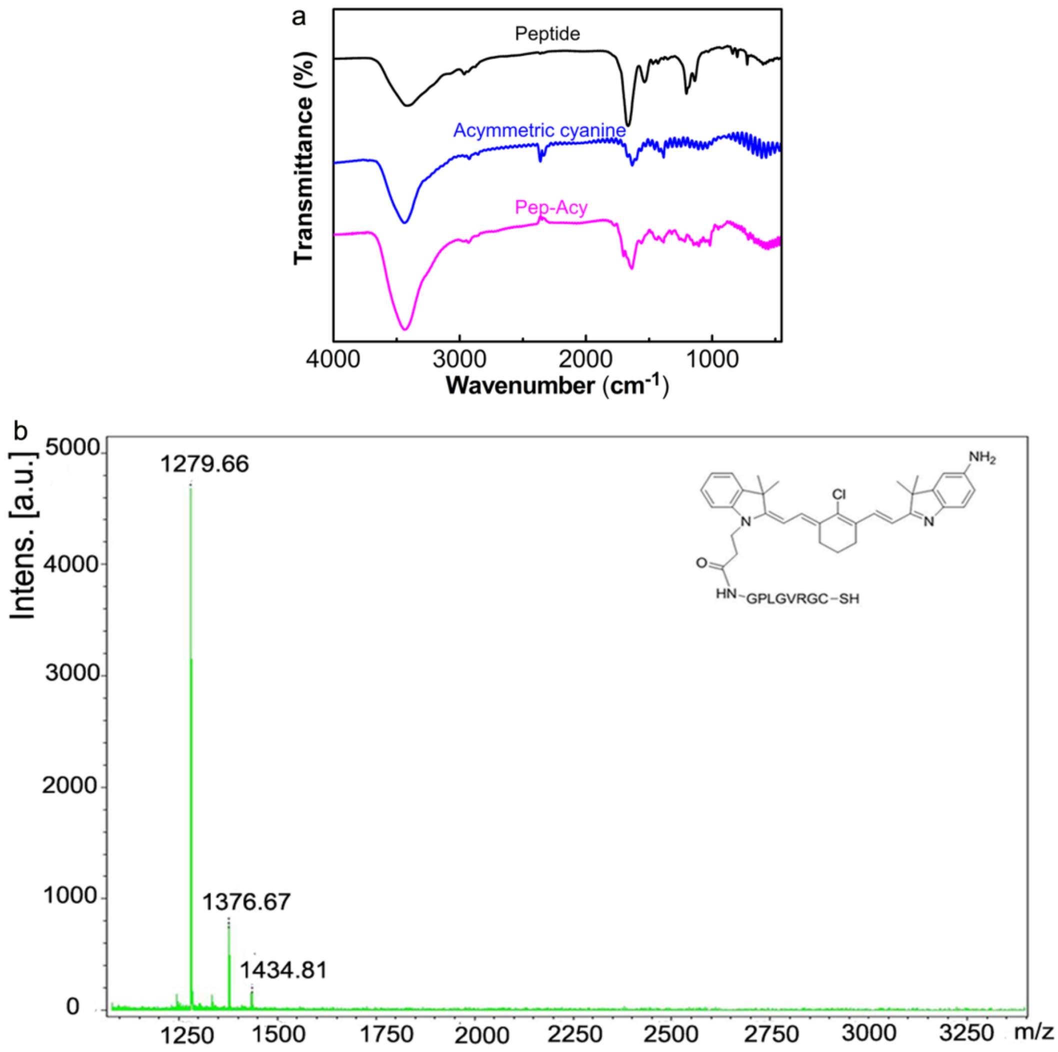

Supplementary Figure 1 | Characterization of the formation of Pep-Acy. a: FT-IR spectra of the asymmetric cyanine, the peptide H_2N-GPLGVRGC-SH and Pep-Acy. **b:** MS spectrum of Pep-Acy.

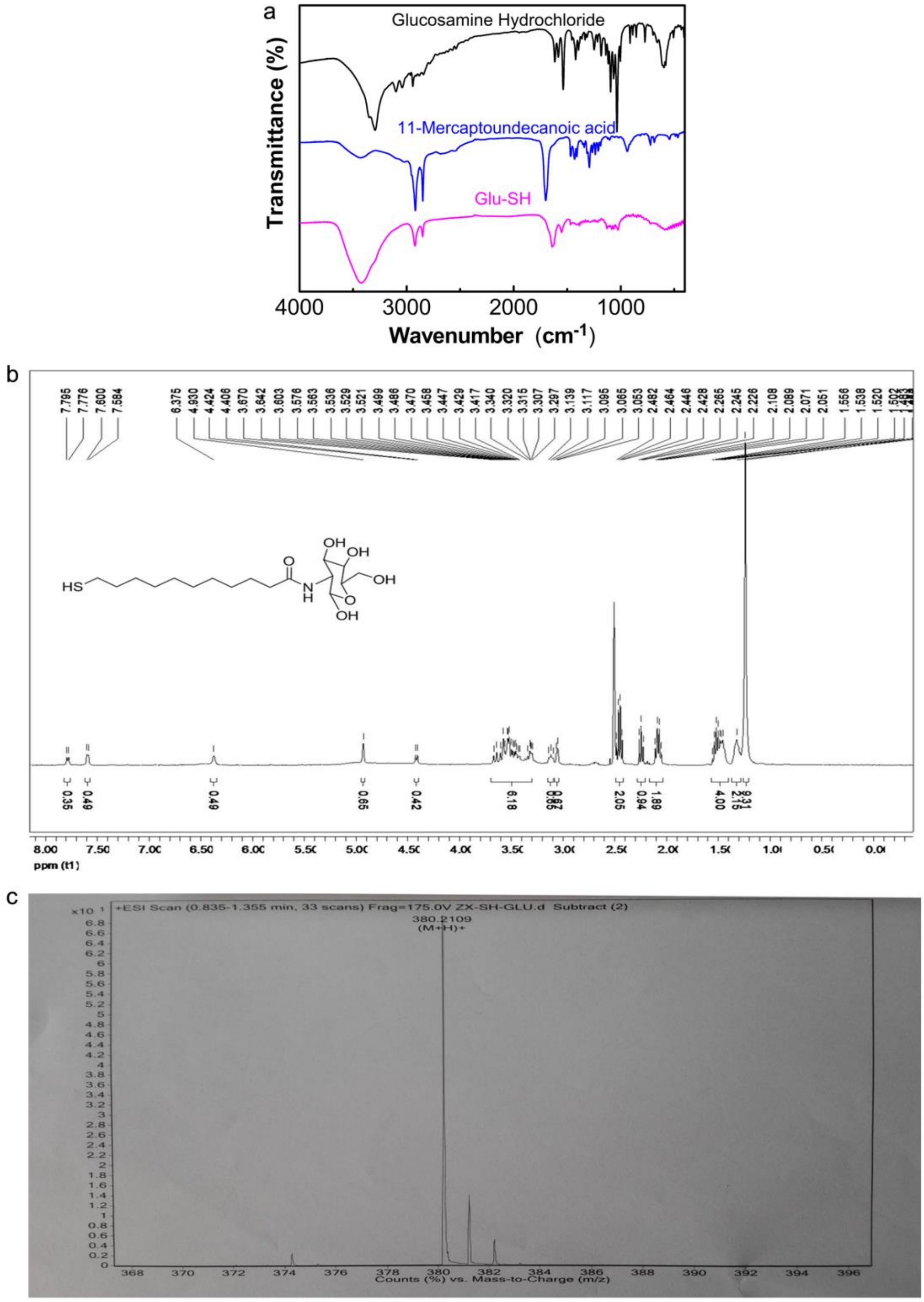

Supplementary Figure 2 | Characterization of the formation of Glu-SH. a: FT-IR spectra of glucosamine hydrodhloride, 11-mecaptoundecanoic acid and Glu-SH. **b:** ^{1}H NMR spectrum of Glu-SH. **c:** HRMS spectrum of Glu-SH.

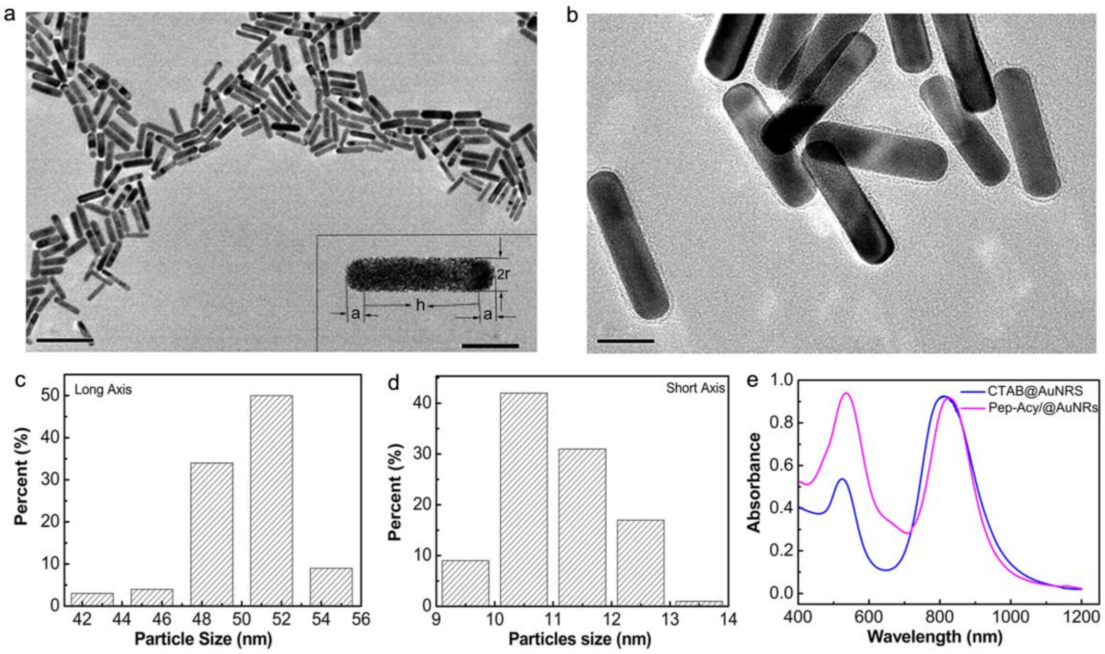

Supplementary Figure 3 | Characterization of AuNRs before and after functionalization. a and b: TEM images of CTAB@AuNRs (Scale bar, 100 nm. Inset is enlarged image and the scale bar is 20 nm) and Pep-Acy@AuNRs (Scale bar, 20 nm), respectively. **c and d:** The particle size distribution of CTAB@AuNRs calculated from 100 randomly selected nanorods in TEM images. **e:** UV-vis-NIR absorption spectra of CTAB@AuNRs and Pep-Acy@AuNRs at pH 7.4.

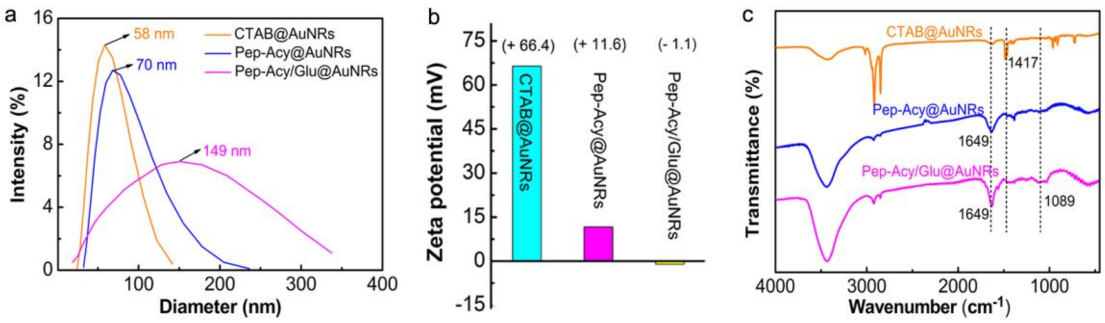

Supplementary Figure 4 | Characterization of AuNRs before and after functionalization. a: Hydrodynamic diameter distribution of CTAB@AuNRs, Pep-Acy@AuNRs and Pep-Acy/Glu@AuNRs. **b:** Zeta potentials of CTAB@AuNRs, Pep-Acy@AuNRs and Pep-Acy/Glu@AuNRs. **c:** FT-IR spectra of CTAB@AuNRs, Pep-Acy@AuNRs and Pep-Acy/Glu@AuNRs.

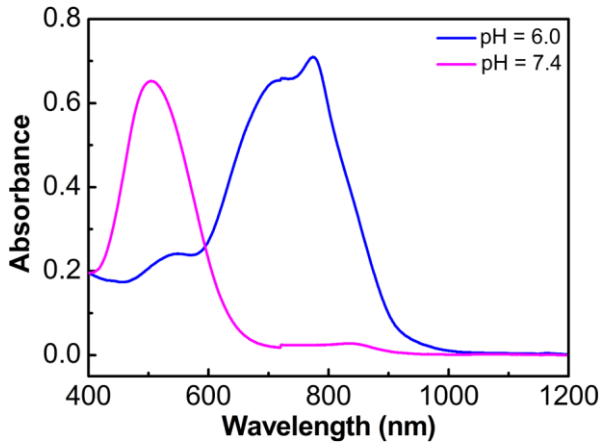

Supplementary Figure 5 | UV-vis-NIR absorption spectra of Pep-Acy at pH 6.0 and 7.4. The prepared Pep-Acy showed a characteristic absorption peak at ca. 525 nm at pH 7.4 (in its base form), and gave a reduced absorption peak at 525 nm and a new absorption peak at ca. 790 nm with a shoulder peak at ca. 700 nm at pH 6.0 due to the transition of the Pep-Acy from its base to acid form.

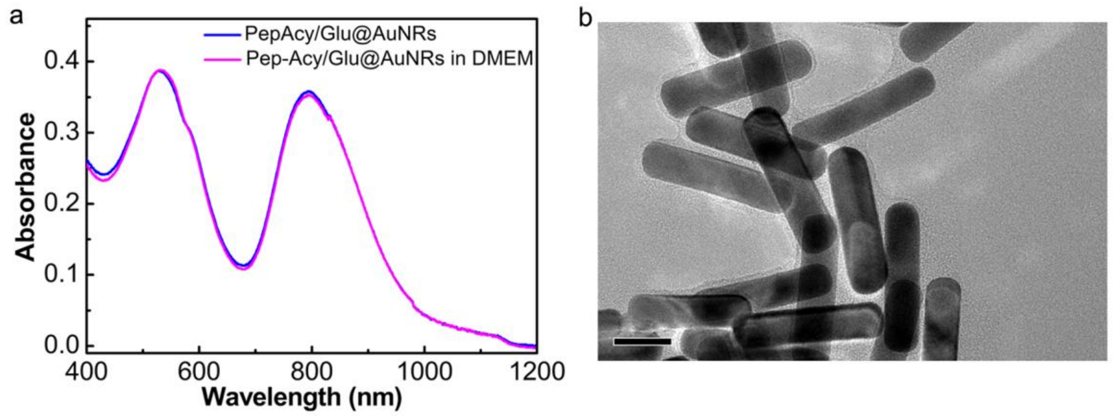

Supplementary Figure 6 | Stability of Pep-Acy/Glu@AuNRs. a: UV-vis-NIR absorption spectra of Pep-Acy/Glu@AuNRs before and after incubation with a complex physiological medium (Dulbecco's modified Eagle's medium (DMEM) with 10% fetal bovine serum (FBS)). **b:** TEM image of Pep-Acy/Glu@AuNRs after incubation with DMEM with 10% FBS (Scale bar, 20 nm).

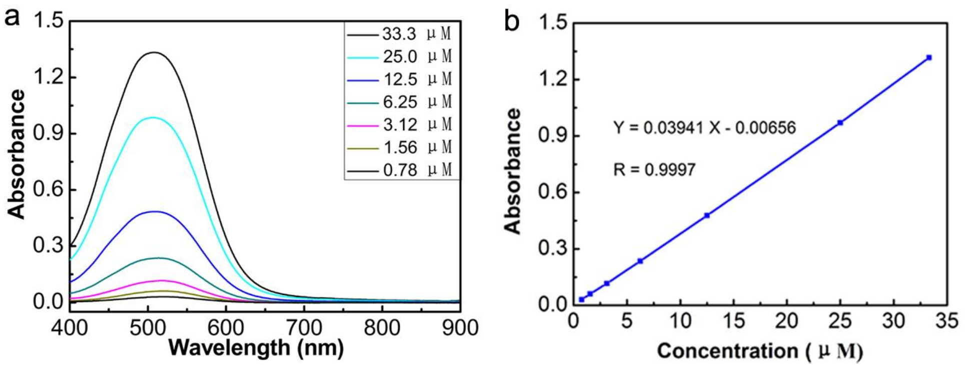

Supplementary Figure 7 | Quantification of Acy on the surface of AuNRs. a: UV-vis absorption spectra of Acy at different concentrations at pH 7.4. **b:** Calibration curve (Absorbance at 525 nm against the concentration of Acy**)** for quantification of Acy at pH 7.4.

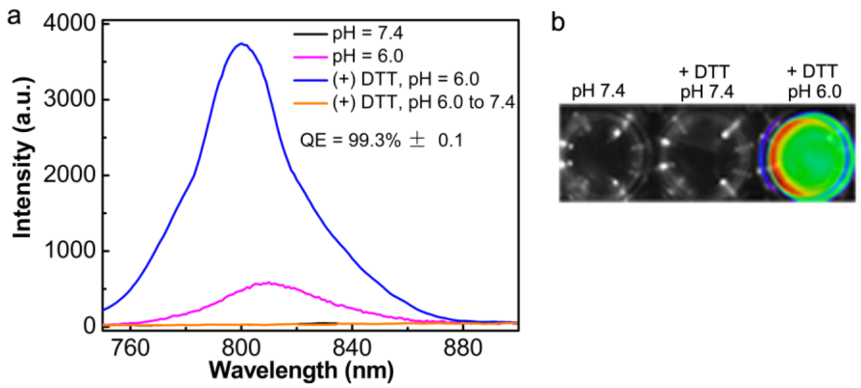

Supplementary Figure 8 | Effect of DTT on the fluorescence of Pep-Acy/Glu@AuNRs. a: Fluorescence spectra of Pep-Acy/Glu@AuNRs before and after adding DTT at pH 6.0 or pH 7.4. **b:** NIR fluorescence imaging of Pep-Acy/Glu@AuNRs before and after adding DTT at different pH values.

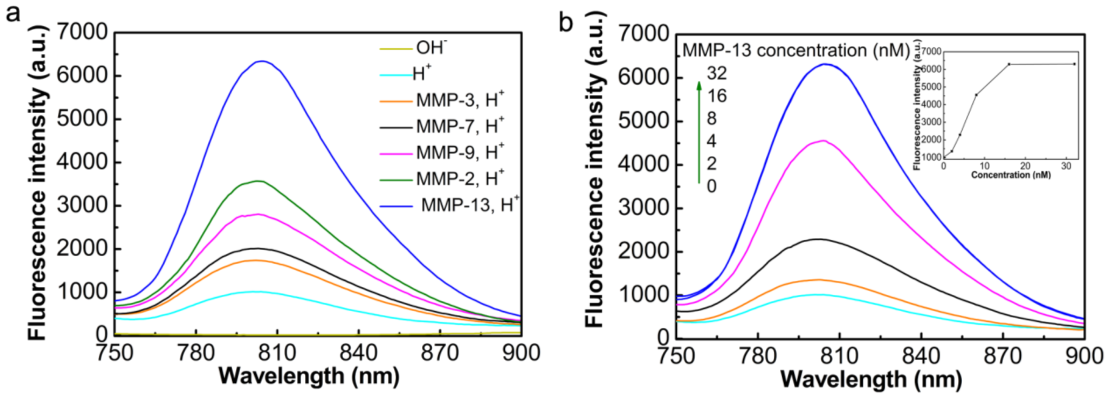

Supplementary Figure 9 | Fluorescence analysis of MMPs activation of Pep-Acy/Glu@AuNRs. a: Responses of the fluorescence spectra of Pep-Acy/Glu@AuNRs (60 μg mL^{-1}, as Au) to multiple types of MMPs (100 nM) and pH (pH 6.0 or 7.4). **b:** Response of the fluorescence spectra of Pep-Acy/Glu@AuNRs (60 μg mL^{-1}, as Au) to various concentrations of MMP-13 at weak acid medium (pH 6.0). Inset: Fluorescence intensity at 805 nm against the concentrations of MMP-13 at pH 6.0.

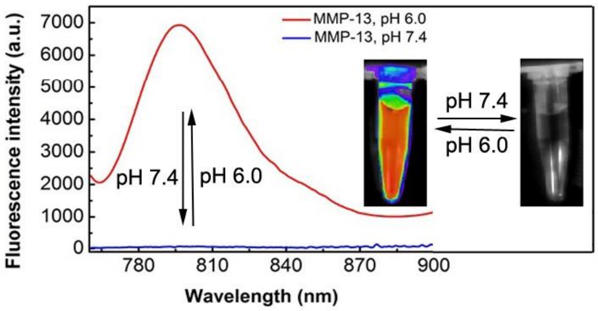

Supplementary Figure 10 | Reversible activation of Pep-Acy/Glu@AuNRs. pH stimulated reversible fluorescence response and fluorescence imaging of Pep-Acy/Glu@AuNRs in the presence of MMP-13.

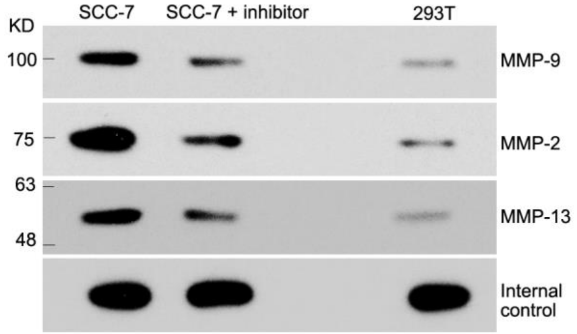

Supplementary Figure 11 | Western blotting Analysis of MMPs expression in the cells. The expression levels of MMP-2, MMP-9 and MMP-13 in 293T and SCC-7 cells pre-treated with or without inhibitor were detected by Western blotting analysis with pressed film method. The results show that 293T low-expressed MMPs, SCC-7 over-expressed MMPs and the expression levels of MMPs were dramatically reduced by inhibitor, in consistent with the results of quantitative MMPs ELISA. The expression levels of MMP-2, MMP-9 and MMP-13 are 822 ± 2.6, 404 ± 3.5 and 235 ± 1.7 pg per 10^7 SCC-7 cells, 70 ± 5.2, 40 ± 4.2 and 32 ± 3.2 pg per 10^7 293 T cells, quantified by ELISA.

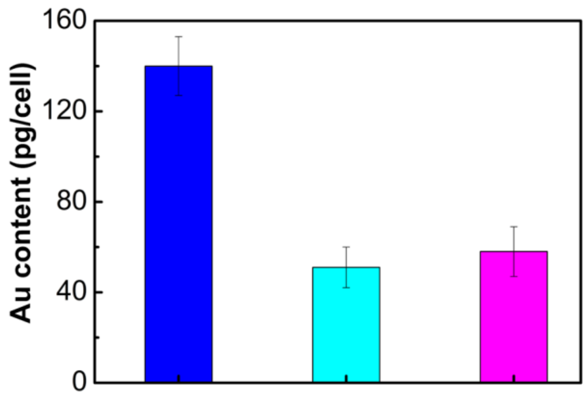

Supplementary Figure 12 | ICP-MS analysis of the intracellular Au. The intracellular Au contents in SCC-7 cells treated with Pep-Acy/Glu@AuNRs (blue) or Pep-Acy@AuNRs (cyan) and glucosamine blocked SCC-7 cells treated with Pep-Acy/Glu@AuNRs (magenta). Error bars are defined as s.d..

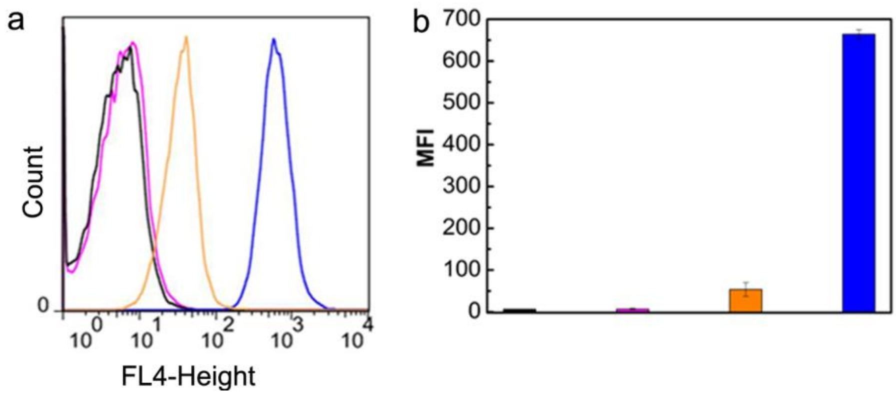

Supplementary Figure 13 | Flow cytometry analysis of SCC-7 cells. a: Flow cytometry profile for the SCC-7 cell incubated with Pep-Acy/Glu@AuNRs under different conditions. Blue and orange: In the absence (blue) or presence (orange) of MMPs inhibitor (low MMPs expressed) at weak acid medium (pH 6.0). Magenta: In the absence of MMPs inhibitor at weak base medium (pH 7.4). Black: without treatment. **b:** Corresponding mean fluorescence intensity (MFI) of SCC-7 cells. The MFI of SCC-7 cells treated with Pep-Acy/Glu@AuNRs at pH 6.0 was much higher than others, indicating that the fluorescence activation of Pep-Acy/Glu@AuNRs was pH and MMPs dependent. Error bars are defined as s.d..

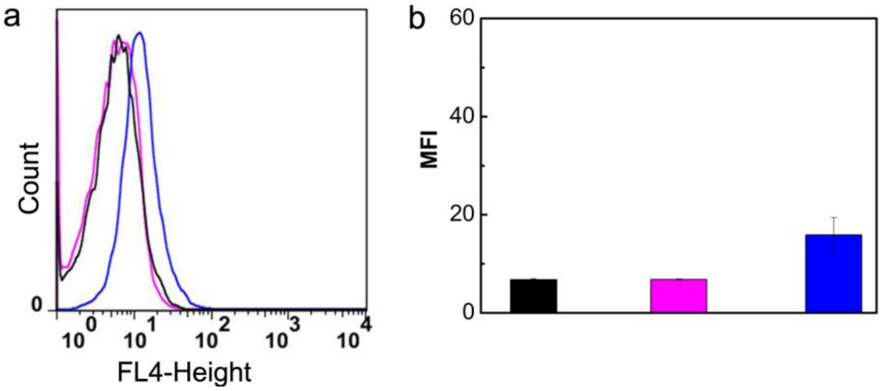

Supplementary Figure 14 | Flow cytometry analysis of MMPs negative 293T cells. a: Flow cytometry profile for the 293T cells incubated with Pep-Acy/Glu@AuNRs. **b:** Corresponding MFI of 293T cells. Blue: weak acid medium (pH 6.0, blue); Magenta: weak base medium (pH 7.4,); Black: without treatment. 293T cells exhibited almost no fluorescence at pH 6.0 although the same amount of Pep-Acy/Glu@AuNRs was applied (compared with Supplementary Figure 17), showing a strong MMPs dependent fluorescence activation. Error bars are defined as s.d..

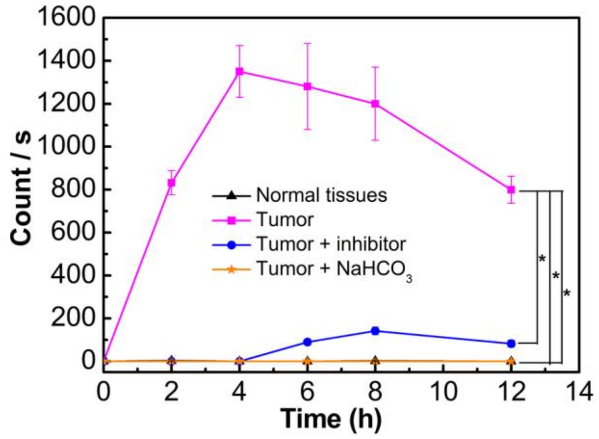

Supplementary Figure 15 | Quantitative analysis of *the in vivo* fluorescence imaging. Pep-Acy/Glu@AuNRs mediated *in vivo* fluorescence imaging was analyzed by counting the number of photos per second in the tumor region treated or untreated with MMPs inhibitor or $NaHCO_3$ and the normal tissues (*P < 0.05). Center values and error bars are defined as mean and s.d., respectively. Statistical significance is assessed by two-way ANOVA

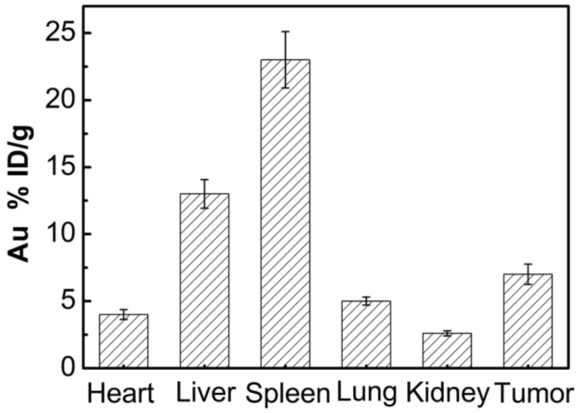

Supplementary Figure 16 | Biodistribution of Pep-Acy/Glu@AuNRs. The biodistribution of Pep-Acy/Glu@AuNRs was analyzed by measuring the Au content in SCC-7 tumor-bearing mice at 12 h post-injection (% ID means the percentage of the injected dose) by ICP-MS. Error bars are defined as s.d..

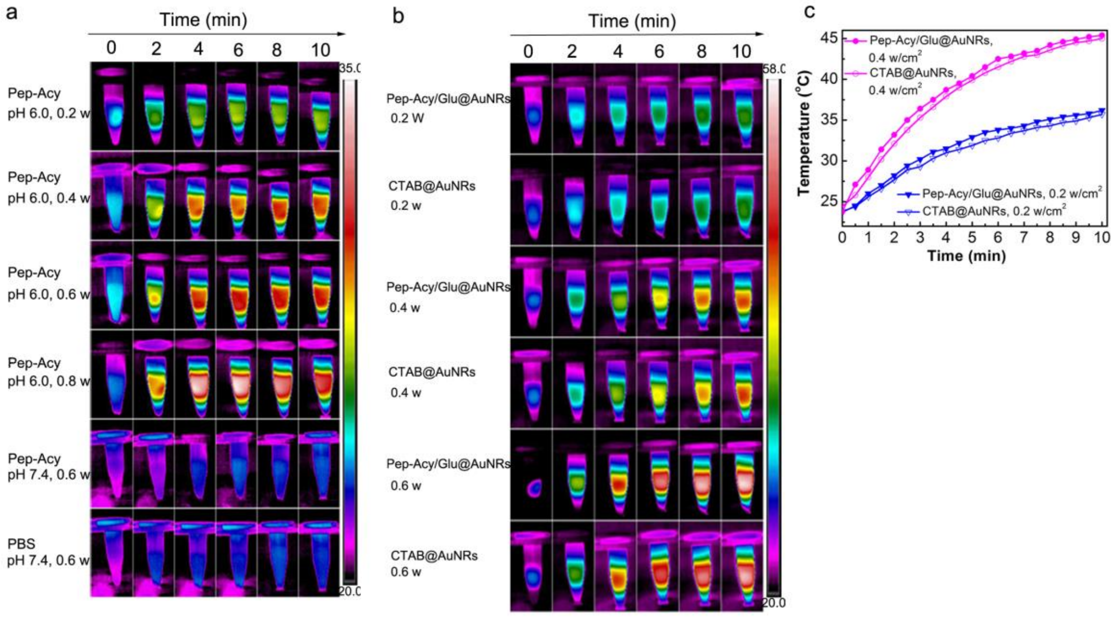

Supplementary Figure 17 | ***In vitro*** **photothermal effect. a:** Representative thermal images of Pep-Acy and PBS with 808 nm laser irradiation. **b:** Representative thermal images of Pep-Acy/Glu@AuNRs and CTAB@AuNRs under 808 nm laser irradiation. **c:** Temperature change curves of Pep-Acy/Glu@AuNRs and CTAB@AuNRs with 808 nm laser irradiation.

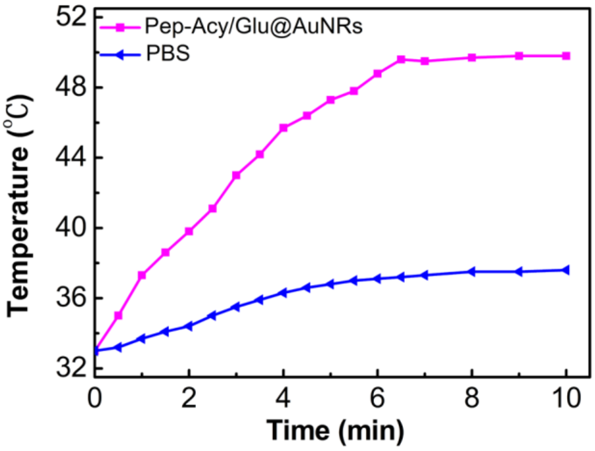

Supplementary Figure 18 | *In vivo* photothermal effect. Temperature change of mice (tumor sites) subjected to 808 nm laser irradiation for 4 h after intravenous injection of Pep-Acy/Glu@AuNRs and PBS.

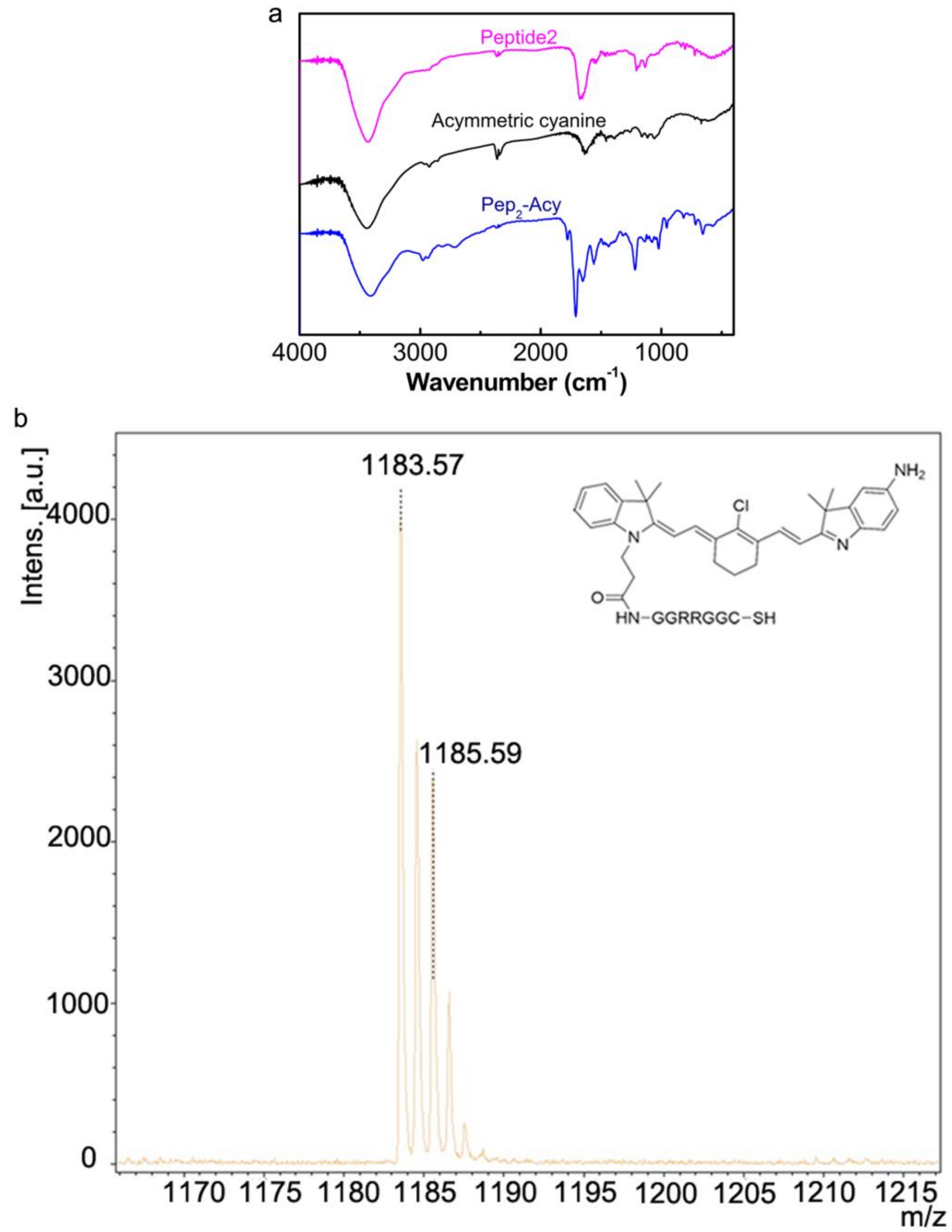

Supplementary Figure 19 | Characterization of the formation of Pep_2-Acy. a: FT-IR spectra of asymmetric cyanine, peptide 2 (H_2N-GGRRGGC-SH) and Pep_2-Acy. **b:** MS spectrum of Pep_2-Acy.

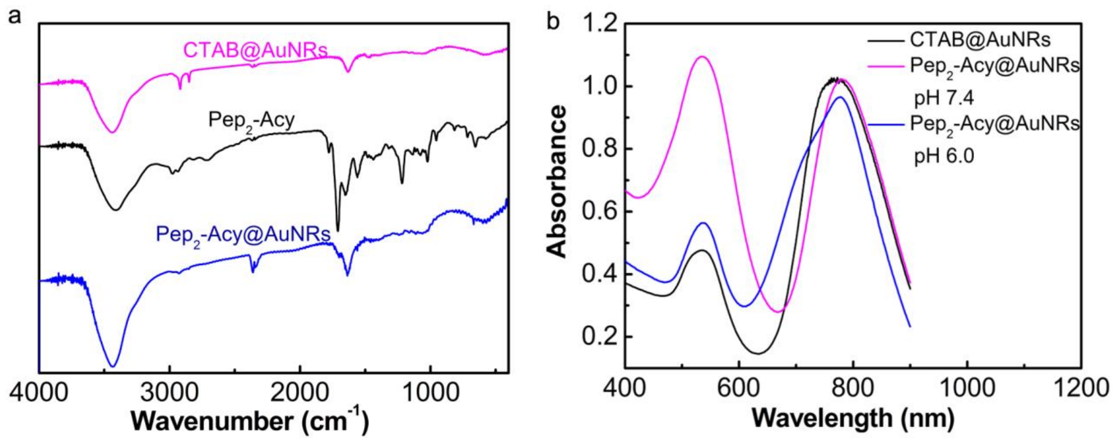

Supplementary Figure 20 | Characterization of Pep$_2$-Acy@AuNRs. a: FT-IR spectra of CTAB@AuNRs, Pep$_2$-Acy and Pep$_2$-Acy@AuNRs**. b:** UV-vis-NIR absorption spectra of Pep$_2$-Acy@AuNRs and CTAB@AuNRs.

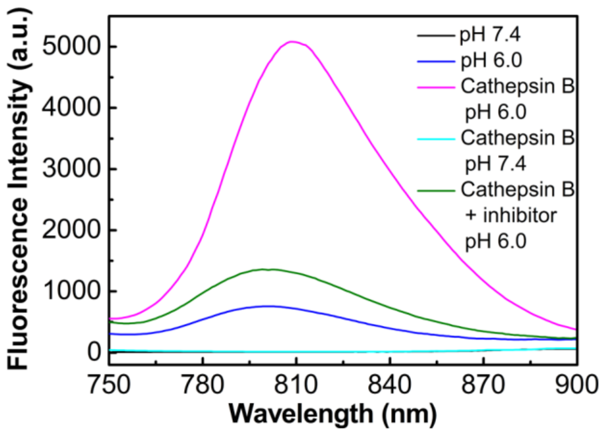

Supplementary Figure 21 | Cathepsin B and pH dependent fluorescence of Pep$_2$-Acy@AuNRs. Fluorescence response of Pep$_2$-Acy@AuNRs (60 μg mL^{-1}, as Au) to cathepsin B and pH with or without inhibitor. Incubating of Pep$_2$-Acy@AuNRs and cathepsin B with simultaneous adjusting pH to 6.0 (simulated tumor acidic microenvironment) induced significant NIR fluorescence signal, while the fluorescence signals was dramaticlly inhibited with cathepsin B inhibitor. Besides, no fluorescence of Pep$_2$-Acy/Glu@AuNRs was lightened with cathepsin B in weak basic medium (pH 7.4, normal biological fluid pH). The above results indicate that the activation of Pep$_2$-Acy@AuNRs should be carried out with synergetic stimulation of acidic microenvironment and cathepsin B.

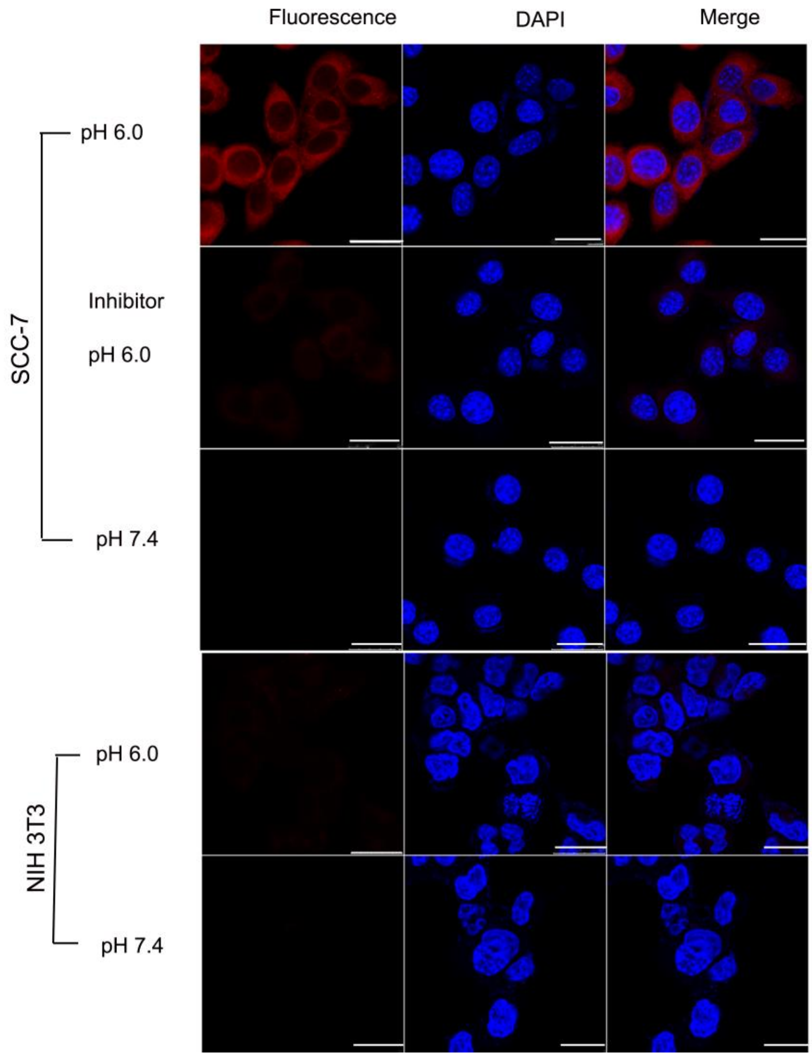

Supplementary Figure 22 | Cell imaging of Pep_2-Acy@AuNRs. Scale bar, 25 µm**.** SCC-7 cells (cathepsin B positive) incubated with Pep_2-Acy@AuNRs at pH 6.0 gave remarkable fluorescence. In comparison, the SCC-7 cells pretreated with MMPs inhibitor (low cathepsin B expressed) showed little fluorescence while NIH 3T3 cells (cathepsin B negative) exhibited almost no fluorescence at pH 6.0 although the same amount of Pep_2-Acy@AuNRs was applied. On the other hand, neither SCC-7 nor NIH 3T3 cells lighted up the fluorescence of Pep_2-Acy@AuNRs at pH 7.4. The above results clearly indicate that the fluorescence of Pep_2-Acy@AuNRs could be switched on if and only if acidic microenvironment and overexpressed cathepsin B co-exist, making it qualified for intelligent tumor-specific imaging.

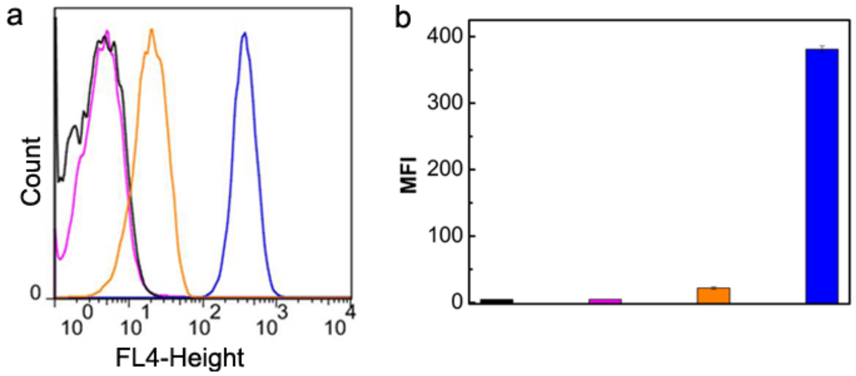

Supplementary Figure 23 | Flow cytometry of SCC-7 cells incubated with Pep$_2$-Acy@AuNRs. a: Flow cytometry profile of SCC-7 cells incubated with Pep$_2$-Acy@AuNRs with or without cathepsin B inhibitor. Blue and Orange: In the presence (blue) or absence (orange) of cathepsin B inhibitor at weak acid medium (pH 6.0). Black: In the absence of cathepsin B inhibitor at weak base medium (pH 7.4). Magenta: Without treatment. b: Corresponding MFI of SCC-7 cells. The MFI of SCC-7 cells treated with Pep$_2$-Acy@AuNRs at pH 6.0 was much higher than others, also indicating that the fluorescence of Pep$_2$-Acy@AuNRs was pH and cathepsin B dependent. Error bars are defined as s.d..

Supplementary methods

Chemicals and materials

Cetyltrimethylammonium bromide (CTAB) was purchased from Sinopharm Chemical Reagent Co. Ltd. (Shanghai, China). $HAuCl_4 \cdot 4H_2O$, $AgNO_3$, $NaBH_4$ and ascorbic acid (AA) were obtained from Guangfu Fine Chemical Research Institute (Tianjin, China). *D*-glucosamine hydrochloride, 1-ethyl-3-(3-dimethylaminopropyl) carbodiimide hydrochloride (EDC·HCl), *N*-hydroxysuccinimide (NHS) and *N,N*-diisopropylethylamine (DIPEA) were bought from Aladdin Reagent Co. Ltd. (Shanghai, China). 3-(4,5-Dimethyl-thiazol-2-yl)-2,5-diphenyltetrazolium bromide (MTT), 11-Mercaptoundecanoic acid and *p*-aminophenol mercuric acid (APMA) were provided by Sigma-Aldrich (Tianjin, China). *DL*-Dithiothreitol (DTT) was obtained from J&K Scientific Ltd. (Beijing, China). Matrix metalloproteinases (MMPs) substrate (H_2N-GPLGVRGC-SH) and cathepsin B substrate (H_2N-GGRRGGC-SH) were supplied by China Peptides Co. Ltd. (Shanghai, China). Recombinant human MMPs (including MMP-2, MMP-3, MMP-7, MMP-9 and MMP-13) proteins, MMP-13 inhibitor (WAY 170523), broad-spectrum MMP inhibitor (ONO 4817) and Recombinant human cathepsin B protein were purchased from R&D Systems (Minneapolis, MN, USA). Cathepsin B inhibitor (VBY-825) was obtained from MedChem express Co., Ltd (Shanghai, China). Quantitative MMPs ELISA kits were purchased from Cloud-Clone Corp. (Wuhan, China). MMP-2 (D8N9Y) Rabbit mAb and MMP-9 (D603H) XP® Rabbit mAb were obtained from Cell Singnaling Technology, Inc. (Shanghai, china). Anti-MMP13 antibody (EP 1263Y) was provided by Abcam Trading (Shanghai) Company Ltd. (Shanghai, china). All the other reagents and solvents were analytical grade and used without further purification unless otherwise noted. Dimethyl sulfoxide (DMSO) was purified by refluxing with calcium hydride for 4 h and then distilled. High-purity

water (Hangzhou Wahaha Group Co. Ltd., Hangzhou, China) was used throughout this work.

Instrumentation and characterization

^1H NMR analysis was carried out on a Bruker Avance 400 spectrometer with tetramethyl silane (TMS) as an internal standard (Bruker, Switzerland). High-resolution mass spectra were recorded on a VG ZAB-HS mass spectrometer (VG Instruments, UK). Mass spectra were measured on an Autoflex III LRF 200-CID (Bruker Daltonics, Germany). Fourier transform infrared spectroscopy (FT-IR) spectra were carried out on a Nicolet 6700 spectrometer (Thermo Fisher Scientific, USA) using a KBr pellet. The absorption spectra were performed on a UV 3600 UV-vis-NIR spectrophotometer (Shimadzu Co., Japan). The fluorescence spectra were acquired on an F-4500 spectrofluorometer (Hitachi, Japan). The transmission electron microscopy (TEM) images were performed on a Tecnai G2 F20 field emission transmission electron microscope (FEI, US) with an accelerating voltage of 200 KV. Dynamic light scattering (DLS) and zeta potential analysis were performed on a Nano-ZS Zetasizer (Malvern, UK). The content of Au in the solution of AuNRs was determined on a 180-80 polarized Zeeman atomic absorption spectrophotometer (AAS) (HITACHI, Japan). The content of gold in cells, major organs and tumor tissue was measured on an X series inductively coupled plasma mass spectrometer (ICP-MS) (Thermo Elemental, UK). MTT assay was performed on a microplate reader (Synergy HT, BioTek). The cell imaging was carried on a TCS SP8 laser confocal scanning microscope (Leica, Germany). Flow cytometry analysis was carried out on FACScan cytometer (BD Biosciences, USA). *In vivo* fluorescence imaging was performed on an IVIS Lumina II imaging system (Xenogen, USA).

Preparation of CTAB@AuNRs

CTAB@AuNRs was prepared according to Wang et al. with a slight modification[1]. Briefly, the seed

solution was prepared by adding freshly prepared and ice-cold $NaBH_4$ (0.6 mL, 10.0 mM) to a mixture, made of CTAB (5.0 mL, 0.2 M) and $HAuCl_4$ (5.0 mL, 0.5 mM). After vigorous stirring for 5 min, the resulting solution was kept at 28 ^{o}C for 4 h before use. Meanwhile, the growth solution was obtained by sequential addition of $HAuCl_4$ (1.8 mL, 5.0 mM), $AgNO_3$ (23 µL, 0.1 M), HCl (20 µL, 1.2 M) and AA (1050 µL, 10.0 mM) into the solution of CTAB (9 mL, 0.2 M) under gentle stirring. Then, 12 µL of seed solution was injected into the mixture rapidly. The resulting solution was stirred for 1 min and then left at 28 ^{o}C overnight for growing the CTAB@AuNRs.

Synthesis of H_2N-GPLGVRGC-SH peptide modified asymmetric cyanine (Pep-Acy)

To a DMSO solution of the asymmetric cyanine (Acy) prepared according to our previous work[2] (54.20 mg, 0.10 mmol), EDC·HCl (28.09 mg, 0.11 mmol) and NHS (12.66 mg, 0.11 mmol) were added and then the resulting mixture was stirred for 12 h at room temperature under N_2 atmosphere. After that, DIPEA (55 µL, 0.33 mmol) and H_2N-GPLGVRGC-SH (84.74 mg, 0.11 mmol) were added and stirred for an additional 8 h at room temperature in the dark. The mixture was poured into diethyl ether (150 mL). The precipitation was collected by filtration and washed three times with diethyl ether and acetone to obtain Pep-Acy as a black powder in 69.1 % yield. FT-IR (powder): 3441, 2926, 2855, 1701, 1657, 1648, 1550, 1532, 1444, 1373, 1195, 1124 (cm^{-1}) (Supplementary Fig. 1a). MS: calc. for: $C_{64}H_{89}ClN_{14}O_{10}S$, $[M-H]^-$ 1279.62, found 1279.66 (Supplementary Fig. 1b).

Pep_2-Acy was obtained by the similar method. FT-IR (powder): 3419, 2964, 2934, 1773, 1714, 1654, 1552, 1432, 1215, 1192, 1137 (cm^{-1}) (Supplementary Fig. 19a). MS: calc. for: $C_{56}H_{77}ClN_{16}O_9S$, $[M-H]^-$ 1183.54, found 1183.57 (Supplementary Fig. 19b).

Synthesis of thiol-terminated glucosamine derivative (Glu-SH)

The mixture of NHS (1.27 g, 11.03 mmol) and EDC·HCl (2.10 g, 10.95 mmol) was added to the DMSO solution of 11-mercaptoundecanoic acid (1.06 g, 9.99 mmol) at room temperature under N_2 atmosphere. After stirring for 12 h, *D*-glucosamine hydrochloride (2.16 g, 10.02 mmol) and DIPEA (5.0 mL, 30.25 mmol) were added. The reaction mixture was continually stirred for an additional 8 h, and then poured into water (200 mL). The precipitation was collected by filtration and re-dissolved in 1.0 mol/L NaOH solution (100 mL). After that, the resulting solution was filtered to remove the residue and then acidified to pH 5.0 with 1 mol/L HCl solution. The resulting precipitation was collected by filtration again to obtain Glu-SH as a white powder (3.03 g, 79.7 %). FT-IR (powder): 3416, 2920, 2847, 1637, 1539, 1295, 1088 (cm^{-1}) (Supplementary Fig. 2a). ^1H NMR (400 MHz, DMSO-d_6) δ(ppm)：7.78 (d, 1H), 7.59 (d, 1H), 6.38 (s, 1H), 4.93 (s. 1H), 4.41 (d, 1H), 3.67-3.30 (m, 6H), 3.10 (t, 1H), 3.06 (d, 1H), 2.45 (q, 2H), 2.25 (t, 1H), 2.08 (q, 2H), 1.56-1.46 (m, 4H), 1.32 (t, 2H), 1.24 (s, 9 H) (Supplementary Fig. 2b). HRMS (ESI Positive): calc. for $C_{17}H_{33}NO_6$, $[M+H]^+$ 380.2106, found 380.2109 (Supplementary Fig. 2c).

Preparation of Pep-Acy@AuNRs

10 mL of CTAB@AuNRs solution was first centrifuged twice at 8000 rpm for 5 min to remove the excess CTAB and then re-suspended in the same volume of high-purity water. To the purified CTAB@AuNRs solution, 2 mL anhydrous DMSO solution of Pep-Acy (2 mg, 1.56 μmol) was added dropwise under vigorous stirring. After stirring for 24 at room temperature in the dark, the solution was centrifuged at 8000 rpm for 5 min at room temperature to remove the excess Pep-Acy. The resulting residue was washed three times with DMSO-water (1:9, v/v) to further remove the unbound Pep-Acy, and then re-dispersed in high-purity water to obtain Pep-Acy@AuNRs. The prepared Pep-Acy@AuNRs was kept in dark for further use.

Pep_2-Acy@AuNRs was obtained by the similar method and characterized with FT-IR (Supplementary Fig. 20a) UV-vis-NIR absorption (Supplementary Fig. 20b) and fluorescence spectroscopy (Supplementary Fig. 21).

Preparation of Pep-Acy/Glu@AuNRs

1.56 μmol of Pep-Acy in anhydrous DMSO (2 mL) was dropwise added to the purified CTAB@AuNRs solution under vigorous stirring. After vigorous stirring for 3 h in the dark, a DMSO solution of Glu-SH (1 mg, 2.63 μmol) was added and stirred at room temperature for an additional 12 h, then centrifuged three times at 8000 rpm for 5 min to remove the excess Pep-Acy and Glu-SH. The final product (Pep-Acy/Glu@AuNRs) was re-dispersed in high-purity water and stored at 4 ^{o}C in dark before use.

Quantitative analysis of Pep-Acy on the surface of AuNRs and calculation of the fluorescence quenching efficiency

To determine the concentration of Pep-Acy bound to the surface of AuNRs, UV-vis titration of asymmetric cyanine (Acy) was carried out at pH 7.4 (Supplementary Fig. 7). Then, the solution of Pep-Acy/Glu@AuNRs at 60 μg/mL (as Au, determined by AAS) was treated with DTT (20 mM) and NaCl (4 M) for 30 min (the strong binding ability of DTT to AuNRs makes Pep-Acy depart from AuNRs[3-5]), and then the resulting mixture was centrifuged. The supernatant was collected and its absorbance at ca. 525 nm was measured by UV-vis-NIR spectroscopy. The concentration of the Acy in the supernatant was determined with a standard calibration method and then the amount of Pep-Acy in the above Pep-Acy/Glu@AuNRs was further calculated.

Besides, each AuNR can be assumed to a cylindrical middle and two spherical segments[6]. The radius (r) and height (h) of cylindrical middle were 5.56 nm and 40.03 nm, respectively, while

the base radius (r) and height (a) of spherical segment were 5.56 nm and 5.04 nm, respectively (determined by TEM images, Supplementary Fig. 3a). Hence, the volume of the AuNR was calculated as follow: $V = \pi a (3r^2+a^2)/3 + \pi r^2 h = 4508.89\ nm^3$. The amount of Au of each AuNR was determined by the formula: amount of Au/AuNR = $\rho_{Au} V M_{Au}/NA = 5.24 \times 10^7 / NA$, Where $\rho_{Au} = 59$ atoms/nm^3, is the density of gold atoms in the bulk; $M_{Au} = 197$ g/mol, is the molar mass of Au. As the amount of Au in the AuNRs solution is 60 μg/mL, the number of the gold rods in this solution was calculated to be 1.45×10^{-9} NA/L. Finally, the grafting density of peptide-probe on each AuNR surface was calculated to be 6234.

To calculation of the fluorescence quenching efficiency, the solution of Pep-Acy/Glu@AuNRs (60 μg/mL, as Au) was treated with DTT (20 mM) and NaCl (4 M) for 30 min and adjusted to pH 6.0 to recover the de-quenched state. Then, the fluorescence spectra of Pep-Acy/Glu@AuNRs in its de-quenched state and quenched state (untreated with DTT, pH 7.4) were recorded (Supplementary Fig. 8a and 8b). The fluorescence quenching efficiency (QE) was calculated via the formula: $QE = (1-\beta) \times 100\%$, where β is the ratio of fluorescence intensity of the quenched to completely de-quenched state[5].

Evaluation of cytotoxicity and *in vitro* photothermal therapy

Standard MTT assay was carried out to evaluate the cytotoxicity and *in vitro* photothermal therapy. Briefly, both SCC-7 and 293T cells were separately seeded into a 96-well plate at a density of 1×10^4 cells per well and incubated overnight, then the medium was replaced with fresh medium containing a series of concentration of CTAB@AuNRs, Pep-Acy@AuNRs or Pep-Acy/Glu@AuNRs (7.5, 15, 30, 60 and 120 μg mL^{-1}). Each concentration was done in quintuplicate. The cells incubated with no AuNRs were used as the blank control. After incubation

for 24 h, the medium was removed and fresh medium containing MTT (0.5 mg mL^{-1}) was added to each well followed by incubation for an additional 4 h. Afterwards, the medium was removed and rinsed with cold PBS, then 100 µL DMSO was added to each well to dissolve the formazan crystals generated by living cells. Finally, the absorbance at 570 nm of each well was recorded on a microplate reader before the plate was vibrated for 20 min in the dark. The relative cell viability (%) was then calculated by the following formula: viability (%) = (the average absorbance of test group / the average absorbance of the blank control) × 100.

For photothermal therapy, SCC-7 cells were seeded in 96-well plate at 1×10^4 cells per well and incubated for 24 h until adherence, then incubated with Pep-Acy/Glu@AuNRs (60 µg mL^{-1}) for an additional 12 h. After that, the cells in each well were subjected to irradiation with an 808 nm laser at various power densities (0.2, 0.4 and 0.6 w cm^{-2}) for different times (3, 6 and 10 min). The cells irradiated under the same conditions but without AuNRs were employed as blank controls. Each experiment group was done in quintuplicate as well. After irradiation, the cells were further incubated for 12 h and then the cell viability was evaluated following the similar procedure mentioned above.

Supplementary References

1. Wang, J. H. *et al*. Bimodal optical diagnostics of oral cancer based on rose Bengal conjugated gold nanorod platform. *Biomaterials* **34**, 4274-4283 (2013).

2. Zhao, X. et al.. A near-infrared multifunctional fluorescent probe with an inherent tumor-targeting property for bioimaging. *Chem. Commun.* **51**, 11721-11724 (2015).

3. Yi, D. K. *et al*. Matrix metalloproteinase sensitive gold nanorod for simultaneous bioimaging and photothermal therapy of cancer. *Bioconjugate Chem.* 2**1**, 2173-2177 (2010).

4. Sun, I. C. *et al*. Tumor-targeting gold particles for dual computed tomography/optical cancer imaging. *Angew. Chem. Int. Ed.* **50**, 9348-9351 (2011).

5. Lee, S. *et al*. A near-infrared-fluorescence-quenched gold-nanoparticle imaging probe for in vivo drug screening and protease activity determination. *Angew. Chem. Int. Ed.* **47**, 2804-2807 (2008).

6. Vigderman, L., Manna, P. & Zubarev, E. R. Quantitative replacement of cetyl trimethylammonium bromide by cationic thiol ligands on the surface of gold nanorods and their extremely large uptake by cancer cells. *Angew. Chem. Int. Ed.* **51**, 636-641 (2012).
